# Shortened leukocyte telomere length as a potential biomarker for predicting the progression of atrial fibrillation from paroxysm to persistence in the short-term

**DOI:** 10.1097/MD.0000000000026020

**Published:** 2021-06-11

**Authors:** Siyu Wang, Yuanfeng Gao, Lei Zhao, Roumu Hu, Xinchun Yang, Ye Liu

**Affiliations:** a Department of Cardiology, Beijing Chao-Yang Hospital, Capital Medical University; b Beijing Key Laboratory of Hypertension, Beijing, China.

**Keywords:** atrial fibrillation, biomarker, risk factors, telomere genetics

## Abstract

This study aimed to assess the role of leukocyte telomere length (LTL) in the development of atrial fibrillation (AF) among Chinese patients.

This is a cross-sectional study. A total of 350 patients from June 2016 to December 2017 were retrospectively analyzed. These included 219 AF patients and 131 with sinus rhythm in the control group. Quantitative real-time PCR was used to measure relative LTL.

The relative LTLs of all subjects (n = 350) ranged from 0.4 to 2.41 (0.98 ± 0.29), showing a significant negative correlation (*P* < .001) with age. The AF-group had significantly shorter LTLs (0.93 ± 0.26 vs 1.07 ± 0.33, *P* < .001) and were older (61.50 ± 6.49 vs 59.95 ± 6.17, *P* = .028) than controls. LTLs among patients with persistent AF (PsAF), paroxysmal AF (PAF), and controls were significantly different (*P* < .001), with LTLs of PsAF patients being the shortest and controls being the longest. After adjusting for possible confounding factors, the PsAF group still showed significantly shorter LTLs than the PAF and control groups (*P* = .013 and *P* = .001, respectively). After an 18-month follow-up, 20 out of 119 PAF patients had progressed into PsAF and a relative LTL of ≤0.73 was an independent predictor for progression of PAF into PsAF.

LTL was found to be shorter in patients with AF than in age-matched individuals with sinus rhythm and positively correlated with severity of AF. LTL shortening could be an independent risk factor for progression from paroxysmal AF to persistent AF in the short term.

## 1 Introduction

Atrial fibrillation is the most prevalent form of arrhythmia worldwide, contributing to an extensive economic and public health burden.^[1]^ This substantial morbidity is associated with increased risk of heart failure, stroke, cardiovascular events, and total mortality.^[2]^ Aging is one of the strongest risk factors for atrial fibrillation (AF), with the prevalence of AF in subjects aged <55 years being <0.1% and increasing to approximately 10% in 80-year-old population.^[3,4]^

Telomeres are the distal ends of eukaryotic chromosomes and comprise tandem repeats of specific DNA sequences (e.g., TTAGGG).^[5,6]^ These chromosomal caps prevent DNA degradation during replication,^[7]^ which could otherwise cause deleterious chromosomal fusions. Humans have telomere lengths of up to 15 to 20 kb, which shortens withaging because of somatic cell division.^[8]^ For example, telomere length of human peripheral white blood cells has been consistently reported to shorten with aging.^[9,10]^ However, the rate of telomere shortening, termed as telomere attrition, could be accelerated by several genetic and environmental factors.^[11]^ Premature or excessive telomere attrition may trigger DNA damage response, which leads to p53 up-regulation and cell apoptosis.^[12]^ In addition, emerging evidence indicates a correlation between short leukocyte telomere length (LTL) and atherosclerosis, heart failure, and left ventricular hypertrophy,^[13]^ which have long been recognized as age-related cardiovascular diseases. Despite the critical importance of aging in AF, its relationship with LTL is controversial.^[14–17]^ In the present study, we aimed to determine whether there is an association between LTL and AF (both persistent and paroxysmal) using a cross-sectional analysis approach.

## 2 Methods

### 2.1 Study design and subjects

This is an observational case-control study. The institutional review board of Beijing Chaoyang Hospital approved this study and all participants have signed informed consent. The study protocol conforms to the ethical guidelines of the 1957 Declaration of Helsinki, as reflected in a priori approval by the Institution's Human Research Committee. All telomere analyses were carried out by a technician who was blind to clinical patient information.

A total of 568 subjects were enrolled in our hospital from June 2016 to December 2017. The inclusion criteria were as follows: aged 18 and older; clinical confirmed diagnosis of atrial fibrillation; have no medical history of valvular heart disease, cardiomyopathy, or acute myocardial infarction; have signed consent form and agreed with the use of blood sample. Paroxysmal AF (PAF) was determined as AF episodes that were self-terminating or converted within 7 days. Persistent AF (PsAF) was classified as AF that lasted longer than 7 days, including episodes terminated by cardioversion after 7 days or more. Concurrently, 131 individuals were recruited as non-AF subjects after multiple telephone follow-up conversations and electrocardiogram (ECG)-examinations for 1 year to rule out undetected AF to the greatest extent possible.

### 2.2 Treatment procedures

Treatments of AF included beta-blockers, amiodarone, and catheter ablation AF patients were treated with catheter ablation or antiarrhythmic agents according to their clinical conditions.

Forty two (35.3%) PAF patients and 39 (39%) PsAF patients underwent catheter ablation. All patients had successful isolation of pulmonary veins (PVs), and the superior vena cava was isolated in 58 (71.6%) patients. In addition to pulmonary vein isolation (PVI) and superior vena cava isolation, LA ablations (including complex fractionated electrograms) were performed on 63 (77.8%) patients. The sets of ablation lesions around the contra lateral PVs intersected at the roof or the posterior wall in 57 (70.4%) patients.

### 2.3 Data collection

All participants’ data were collected from electronic medical records which included demographic features; clinical features; blood routine indexes; echocardiography information. Left ventricular dysfunction was determined as ejection fraction <50% on echocardiography. Renal insufficiency was defined as glomerular filtration rate lower than 30 mL/min. Blood samples were drawn into EDTA-containing tubes at administration and were centrifuged within 2 hours. All samples were processed within 4 hours and stored at –80 °C until use.

### 2.4 DNA extraction

QIAamp DNA Blood Midi Kits (Qiagen, Hilden, Germany) were used to manually extract DNA from blood cells after centrifugation of whole blood samples. Red blood cells were lysed and removed by a series of wash steps. The remaining white blood cells were lysed and solubilized protein was removed by precipitation and centrifugation. DNA quality was determined by ultraviolet absorption at 260 nm/280 nm (Nanodrop 2000 Thermo Scientific).

### 2.5 LTL measurement

The genomic DNA of peripheral blood leukocytes was isolated to measure LTL by quantitative polymerase chain reaction, which was modified from the method reported by Cawthon.^[18]^ The telomere repeats (primers: forward-acactaaggtttgggtttgggtttgggtttgggttagtgt; reverse-tgttaggtatccctatccctatccctatccctatccctaaca) of a single gene (*HBG*, hemoglobin; primers: forward-cttcatccacgttcaccttg; reverse-gaggagaagtctgccgtt) in each sample were measured using quantitative real-time PCR. The relative telomere length was calculated as the ratio of telomere DNA repeats to single-copy gene (SCG) copies (*t*/*s* ratio), with *HBG* being designated as the single-copy gene. All rtPCR experiments were conducted in triplicate for each sample.

### 2.6 Follow-up approaches

From Jun 2016 to Dec 2017, all PAF patients and sinus rhythm controls were routinely followed-up with at 3, 6, 12, and 18 months through outpatient clinic attendance, telephone contact, or review of medical notes. Additionally, unplanned visits were recorded. AF was discovered using ECG records, Holter telemetry, or ECG monitoring devices. 12-lead ECGs or Holter telemetry were arranged at each visit. ECG and Holter results were reported by patients if they were not able to return to our outpatient department. Follow-up patients admitted in our department were monitored through an in-hospital long-term ECG monitoring device or examination of patient history in combination with ECG confirmation during regular or unplanned visits. New AF cases in the sinus rhythm group or progression of PAF into PsAF were used as endpoints. Occurrences of new onset AF or progressions of PAF into PsAF were documented. New onset AF was defined as recorded AF on an ECG in sinus rhythm patients. Progression of paroxysmal AF into persistent was defined as: reported AF symptoms and confirmation by ECG or Holter telemetry which lasted >7 days at visits or visited with an AF state (likewise confirmed by ECG or Holter telemetry) but did not convert within 7 days after the onset of symptoms.

### 2.7 Statistical analysis

Continuous variables were expressed as mean or median ± standard deviation for normally distributed and non-normally distributed ones, separately. Numerical differences between 2 groups were assessed by chi-square test for categorical variables, and *t* test or one-way analysis of variance (ANOVA) for normally distributed continuous variables, while Wilcoxon rank-sum test for non-normally distributed variables. Variables in univariate analysis with *P* value <.1 were further included in multivariate analysis. Logistic regression was used to compare LTL among groups after adjusting for potential confounders including body mass index (BMI), heart rate, sex, history of hypertension and diabetes, left ventricular dysfunction, high sensitive C-reactive protein (hsCRP), LA diameter, and the use of statins and renin angiotensin aldosterone system (RAAS) inhibitors. The value of *t*/*s* ratio was negatively transformed.

Receiver operating characteristic (ROC) curves were conducted to evaluate diagnostic effects of telomere length for subtypes of AF. Cut-off values of *t*/*s* were defined as the point with the largest value of specificity + sensitivity and were used for follow-up analysis. Predictors for AF incidence or progression were calculated from a Cox proportional hazards model with confidence intervals of 95% and covariate adjustment. AF progression events (new onset AF developed from sinus rhythm or PsAF developed from PAF) for LTL with predefined values were examined with an unadjusted Kaplan–Meier curve; a log-rank test was used to compared the AF progression events of patients with *t*/*s* values below and above cut-off values. The threshold for significance was *P* = .05. All statistical analyses were conducted using SPSS, Version 23.0 (SPSS Inc., Chicago, IL).

## 3 Results

### 3.1 Characteristics of the studied subjects

**Figure 1:**
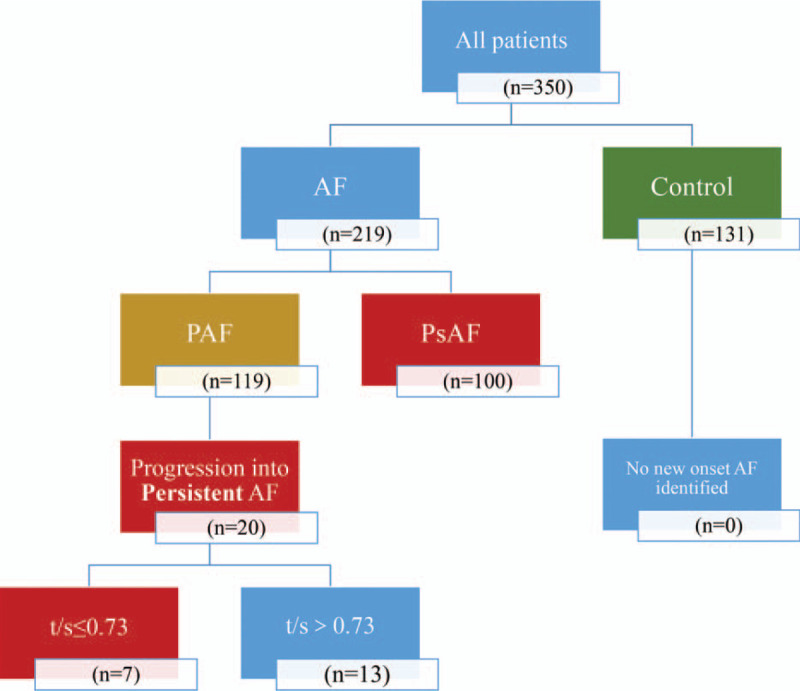
Process of the patient flow diagram.

A total of 568 AF patients and 131 controls were included in this study. Three hundred fifty were eventually finished follow-up and enrolled for further analysis, including 219 AF patients (119 PAF cases and 100 PsAF cases) and 131 controls. Patient flow diagram of all participants was illustrated in Fig. 1.

The mean age was 60.92 years (ranges from 42 to 85) and there were 207 men (59.1%) and 143 women (40.9%). Demographic characteristics were displayed in Table 1. The mean LTLs of all subjects was 0.98 (ranges from 0.40 to 2.41), and AF patients had significant shorter LTL than non-AF patients with *P* value <.001, Besides, there were statistical differences in age (*P* = .007), sex (*P* = .043), heart rate (*P* < .001), presence of hypertension (*P* = .005), and diabetes (*P* = .005), history of left ventricular dysfunction (*P* = .013), RAAS inhibitors (*P* = .001), and statins (*P* = .023) intaken between the 2 groups. No other significant difference was found.

**Table 1:** Demographics between AF and non-AF groups.

Parameters	AF (n = 219)	Non-AF (n = 131)	*P* value
*t*/*s*	0.91 (0.78–1.07)	1.01 (0.84–1.18)	<.001
Age, y	62 (57–67)	60 (56–64)	.007
Gender (male/female)	139/80	68/63	.043
BMI, kg/m^2^	26.29 (24.51–28.04)	26.29 (24.22–26.29)	.052
Heart rate, bpm	80 (68–100)	70 (62–78)	<.001
Hypertension (n)	139 (63.5%)	63 (48.1%)	.005
Diabetes mellitus (n)	60 (27.4%)	19 (14.5%)	.005
CAD (n)	61 (27.9%)	47 (35.9%)	.122
Hyperlipidemia (n)	117 (53.4%)	68 (51.9%)	.825
LV dysfunction (n)	17 (7.8%)	2 (1.5%)	.013
Renal insufficiency (n)	1 (0.5%)	0	.293
Smoke (n)	78 (35.6%)	40 (30.5%)	.352
Alcohol (n)	43 (19.6%)	20 (15.3%)	.319
ACEI/ARB (n)	95 (43.4%)	34 (26.0%)	.001
Statins (n)	123 (56.7%)	91 (69.5%)	.023
β-blockers (n)	104 (47.5%)	59 (45.0%)	.660
LDL, mmol/L	2.60 (2.00–3.10)	2.70 (2.20–3.00)	.574
HsCRP, mg/L	1.49 (0.75–3.67)	1.47 (0.56–2.66)	.278

ACEI = angiotensin converting enzyme inhibitor, ARB = angiotensin receptor blocker, BMI = body mass index, CAD = coronary artery disease, hsCRP = high sensitive C-reactive protein, LV dysfunction = left ventricular dysfunction, LDL = low density lipoprotein.

### 3.2 Independent risk factors of AF

**Figure 2:**
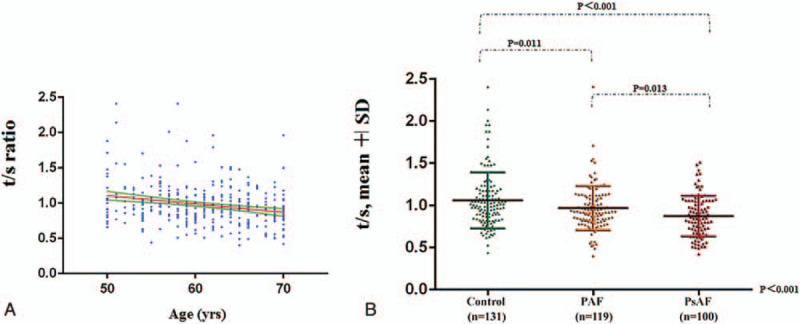
Distribution of LTL in the study. A. Correlation of *t*/*s* ratio and age. Scatter plot showed significant negative correlation (<0.001) between decreasing *t*/*s* and increasing age, which was verified by linear regression. B. Mean telomere length ± SD in 2 AF groups and controls. Differences among groups were significant (0.97 ± 0.26 vs 0.88 ± 0.24 vs 1.07 ± 0.33, *P* < .001). Examination of groups by LSD post hoc test revealed significant differences in all 3 pairwise tests. (paroxysmal AF vs control, *P* = .011; persistent AF vs control, *P* < .001; persistent AF vs paroxysmal AF, *P* = .013). AF = atrial fibrillation, LTL = leukocyte telomere length.

Results from linear regression (Fig. 2A) showed a significant negative correlation (*P* < .001) with age. After adjusting for potential confounders, Table 2 indicated the association between shorter LTLs and presence of AF (OR = 3.51, *P* = .009). Additionally, male patients (OR = 2.52, *P* = .001), heart rate (OR = 1.04, *P* < .001), and diabetes (OR = 2.55, *P* = .006) were independent risk factors (Table 2) of AF. Interestingly, statin usage (OR = 0.47, *P* = .011) acted as a protective factor against AF.

**Table 2:** Independent risk factors of AF from logistic regression.

Parameters	Odds ratio	Lower 95% CI	Upper 95% CI	*P* value
Shorter LTL	3.51	1.38	8.96	.009
Age	1.03	0.99	1.07	.204
Male	2.52	1.47	4.33	.001
BMI	1.03	0.95	1.12	.417
Heart rate	1.04	1.03	1.06	<.001
Hypertension	1.52	0.85	2.72	.160
Diabetes mellitus	2.55	1.31	4.94	.006
CAD	0.72	0.40	1.30	.276
LV dysfunction	4.58	0.90	23.24	.066
ACEI/ARB	1.36	0.73	2.54	.337
Statins	0.47	0.26	0.84	.011
hsCRP	1.00	0.92	1.09	.981

ACEI = angiotensin converting enzyme inhibitor, ARB = angiotensin receptor blocker, BMI = body mass index, CAD = coronary artery disease, hsCRP = high sensitive C-reactive protein, LV dysfunction = left ventricular dysfunction.

### 3.3 Comparison of demographic characteristics between 2 types of AF and controls

Among the 219 AF patients, 119 met the criteria of paroxysmal AF, while 100 met the criteria of persistent AF. Demographic characteristics of 2 AF groups as well as the control group are listed in Table 3. There were statistical differences in age (*P* = .013), sex (*P* = .033), heart rate (*P* < .001), presence of hypertension (*P* = .015), and diabetes (*P* = .015), history of left ventricular dysfunction (*P* < .001), RAAS inhibitors (*P* = .004), and statins (*P* = .027) intaken, LA diameter (*P* < .001), and HsCRP (*P* = .004) among the 3 groups. No other significant difference was found, specifically, PsAF group had the shortest LTLs (mean *t*/*s* 0.88), followed by PAF group (mean *t*/*s* 0.97) and control group (mean t/s 1.07) and they were significant different from each other with *P* value <.001 (Table 3 and Fig. 2B).

**Table 3:** Demographic comparison among PAF, PsAF, and non-AF group.

Parameters	PAF (n = 119)	PsAF (n = 100)	Control group (n = 131)	*P* value
*t*/*s*	0.93 (0.82–1.12)	0.86 (0.70–1.06)	1.01 (0.84–1.18)	<.001^∗^
Age, y	61 (57–66)	64 (58–68)	60 (56–64)	.013
Gender (male/female)	81/38	58/42	68/63	.033
BMI, kg/m^2^	26.29 (23.94–26.85)	26.29 (26.27–29.39)	26.29 (24.22–26.29)	<.001
Heart rate, bpm	71 (62–87)	85 (75–100)	70 (62–78)	<.001^∗^
Hypertension (n)	78 (65.5%)	61 (61.0%)	63 (48.1%)	.015
Diabetes mellitus (n)	35 (29.4%)	25 (25.0%)	19 (14.5%)	.015
CAD (n)	27 (22.7%)	34 (34.0%)	47 (35.9%)	.057
Hyperlipidemia (n)	64 (53.8%)	53 (53.0%)	68 (51.9%)	.957
LV dysfunction (n)	2 (1.7%)	15 (15.0%)	2 (1.5%)	<.001
Renal insufficiency (n)	0	1 (1.0%)	0	.285
Smoke (n)	49 (41.2%)	29 (29.0%)	40 (30.5%)	.103
Alcohol (n)	29 (24.4%)	14 (14.0%)	20 (15.3%)	.081
ACEI/ARB (n)	50 (42.0%)	45 (45.0%)	34 (26.4%)	.004
Statins (n)	63 (52.9%)	60 (60.0%)	91 (69.5%)	.027
β-blocker (n)	50 (42.0%)	54 (54.0%)	59 (45.0%)	.189
LA diameter, mm	39.41 ± 5.39	41.58 ± 4.19	35.35 ± 4.22	<.001^∗^
LDL, mmol/L	2.60 (2.00-3.10)	2.50 (1.90-3.10)	2.70 (2.20-3.00)	.819
HsCRP, mg/L	1.24 (0.60-2.28)	2.18 (1.04-5.46)	1.53 (0.62-2.66)	.004

ACEI = angiotensin converting enzyme inhibitor, ARB = angiotensin receptor blocker, BMI = body mass index, CAD = coronary artery disease, hsCRP = high sensitive C-reactive protein, LDL = low density lipoprotein, LV dysfunction = left ventricular dysfunction.

∗ Significant in pairwise LSD post hoc tests.

### 3.4 Shorter telomere length serves as an independent risk factor for persistent AF, but not paroxysmal AF

To further substantiate the association of telomere length and AF subtypes, we performed logistic regression after adjusting for potential confounders. As described in Table 4, LTL of persistent AF group was found to be shorter than both paroxysmal AF and non-AF groups (OR = 6.31, *P* = .013 and OR = 14.73, *P* = .001, respectively). However, the significant difference of LTLs between paroxysmal AF with non-AF individuals disappeared after adjustment (OR = 2.33, *P* = .147).

**Table 4:** Multinomial logistic regression analysis of PAF, PsAF, and non AF controls.

Parameters	Odds ratio	Lower 95% CI	Upper 95% CI	*P* value
PAF versus Controls				
Shorter LTL	2.33	0.94	7.33	.147
Age	0.99	0.94	1.05	.741
BMI	0.84	0.75	0.95	.005
Heart rate	1.03	1.01	1.06	.003
Male	2.05	0.99	4.21	.051
ACEI/ARB	1.27	0.58	2.77	.554
Statins	0.39	0.19	0.80	.011
LV dysfunction	0.53	0.05	5.29	.585
Hypertension	1.58	0.74	3.38	.238
Diabetes mellitus	2.03	0.92	4.48	.081
CAD	1.92	0.90	4.11	.093
hsCRP	0.98	(0.88	1.09	.663
LA diameter	1.21	1.11	1.31	<.001
PsAF versus Control				
Shorter LTL	14.73	3.16	68.62	.001
Age	1.00	0.94	1.06	.979
BMI	1.07	0.96	1.20	.216
Heart rate	1.06	1.03	1.08	<.001
Male	0.86	0.39	1.91	.712
ACEI/ARB	0.98	0.41	2.34	.963
Statins	0.61	0.27	1.40	.242
LV dysfunction	4.30	0.68	27.40	.123
Hypertension	0.50	0.22	1.18	.687
Diabetes mellitus	1.20	0.50	2.92	.684
CAD	1.18	0.52	2.67	.694
hsCRP	1.07	0.96	1.19	.205
LA diameter	1.27	1.16-	1.39	<.001
PsAF versus PAF				
Shorter LTL	6.31	1.46	27.22	.013
Age	1.01	0.95	1.07	.767
BMI	1.27	1.13	1.44	<.001
Heart rate	1.02	1.00	1.04	.030
Male	0.42	0.20	0.88	.022
ACEI/ARB	0.77	0.35	1.68	.518
Statins	1.57	0.76	3.24	.226
LV dysfunction	8.18	1.51	44.44	.015
Hypertension	0.32	0.15	0.70	.004
Diabetes mellitus	0.59	0.27	1.29	.189
CAD	0.61	0.28	1.33	.217
hsCRP	1.09	1.00	1.20	.054
LA diameter	1.05	0.98	1.13	.162

ACEI = angiotensin converting enzyme inhibitor, ARB = angiotensin receptor blocker, BMI = body mass index, CAD = coronary artery disease, hsCRP = high sensitive C-reactive protein.

### 3.5 ROC analysis indicated a discriminating role of LTLs between AF and sinus rhythm

**Figure 3:**
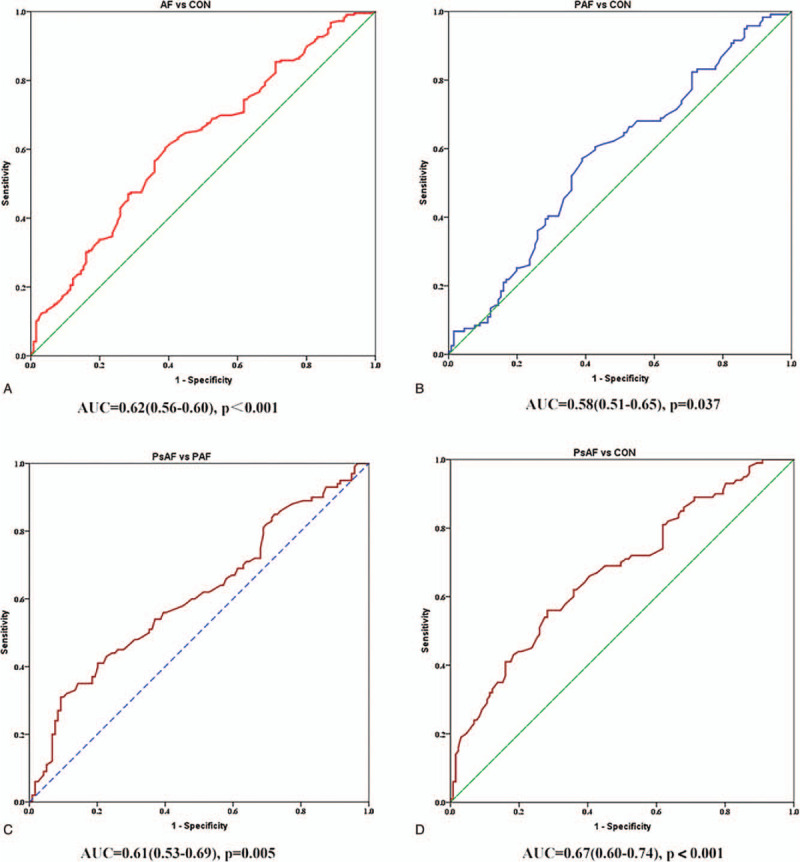
ROC analysis of LTLs in order to distinguish AF and AF subtypes from sinus rhythm. AF = atrial fibrillation, LTL = leukocyte telomere length, ROC = receiver operating characteristic.

From the observation of the significant shorter LTLs in AF patients, especially the PsAF group, we investigated whether shortened LTLs could predict AF onset (defined by development of PsAF or PAF from sinus rhythm) or AF progression (defined by development of PsAF from PAF or sinus rhythm) based on ROC analysis. The results from area under the curve (AUC) analysis indicate that diagnostic value of shorter LTLs represented by *t*/*s* ratios is superior for predicting AF or progression of AF when *P* < .05. As shown in Fig. 3, for total AF versus control, the AUC value was 0.62, *P* < .001. The AUC values were 0.61 and 0.67 for PsAF versus PAF and PsAF versus sinus rhythm control, respectively, with asymptotic significance below 0.05. AUC for PAF versus control was 0.58 with *P* value .037. Cutoff values of LTLs were 0.96 for AF versus sinus rhythm and 0.73 for PsAF versus PAF. These results indicate a potential predictive role of LTL in discriminating PsAF from either PAF or sinus rhythm. Cut-off values were used for the follow-up study.

### 3.6 Follow-up and survival analysis revealed shorter LTL as an independent predictor for progression of PAF to PsAF

**Figure 4:**
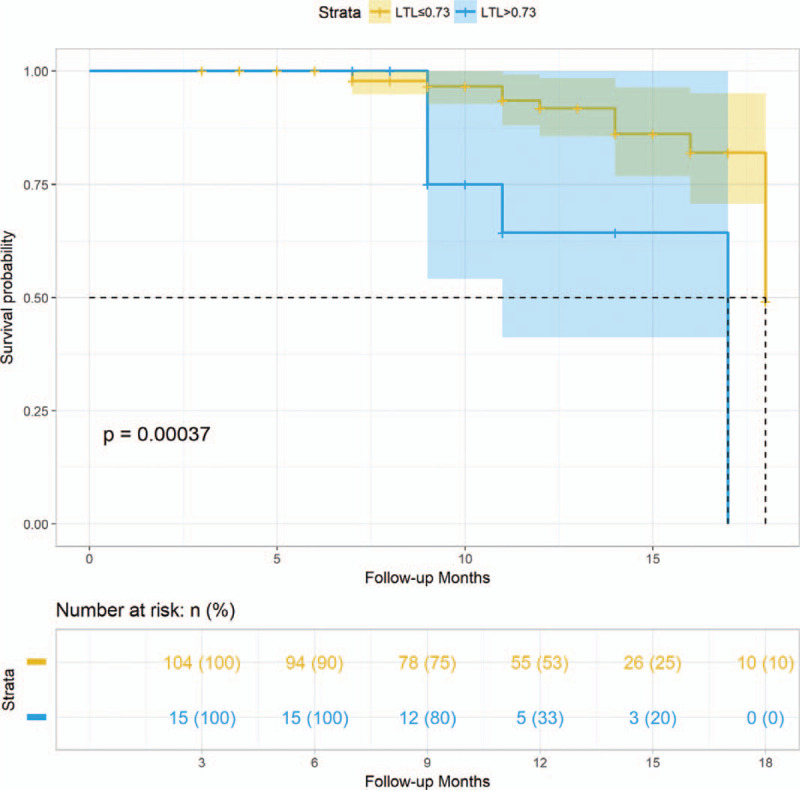
Kaplan–Meier plot of follow-up of PAF patients with a LTL cutoff value of 0.73. LTL = leukocyte telomere length, PAF = paroxysmal atrial fibrillation.

At the end of the follow-up, 20 out of 119 patients primarily diagnosed with paroxysmal AF eventually progressed into persistent AF, while no patient in the control group were newly diagnosed as AF. LTL ≤0.73 (the cut off value that best distinguished persistent AF from paroxysmal AF) was used as the only classified factor for paroxysmal AF patients. Fifteen (14.4%) of 119 paroxysmal AF patients presented with LTL ≤0.73, among which 7 (46.7%) developed persistent AF. As shown in Table 5, grouped analysis based on LTL cut-off *t*/*s* value of 0.73 revealed significant difference in heart rate (*P* = .020). Patients from both groups showed no significant difference in use of β-blocker (*P* = 1), amiodarone (*P* = 0.136), and catheter ablation (*P* = 1). HATCH score was not statistical significant between groups (*P* = 0.501). In a multivariate Cox proportional hazards model, LTL ≤0.73 remained independently significantly related with progression from PAF into PsAF (HR 3.98, 95% CI 1.32–11.94, *P* = .014). Heart rate did not remain significant in multivariate analysis (HR = 0.98, *P* > .05). Kaplan–Meier analysis at 18-month follow-up (Fig. 4) demonstrated that progression of paroxysmal AF into persistence was more likely to occur in patients with LTL ≤0.73 (log rank test 14.56, *P* < .001). For patients who progressed into persistent AF, the median time was 11 months.

**Table 5:** Demographics of paroxysmal AF patients at follow-up.

Parameters	*t*/*s* ≤ 0.73 (n = 15)	*t*/*s* > 0.73 (n = 104)	*P* value
New PsAF	7 (46.7%)	13 (12.5%)	.004
Age, y	63 (60–66)	60 (56–66)	.314
Gender (male/female)	8/7	73/31	.238
BMI	25.08 (22.41–26.29)	26.29 (24.23–27.34)	.248
Heart rate, bpm	66 (55–69)	73 (62–90)	.020
LV dysfunction (n)	0	2 (2%)	1.00
Hypertension (n)	10 (66.7%)	68 (65.4%)	1.00
Diabetes mellitus (n)	5 (33.3%)	30 (28.8%)	.765
CAD (n)	5 (33.3%)	22 (21.2%)	.326
ACEI/ARB (n)	8 (53.3%)	42 (40.4%)	.407
Statins (n)	8 (53.3%)	48 (46.2%)	.783
β-blocker (n)	6 (40%)	44 (42.3%)	1
Amiodarone (n)	8 (53.3%)	76 (73.1%)	.136
HsCRP, mg/L	0.64 (0.35–1.76)	1.29 (0.66–2.55)	.181
LA diameter, mm	39.20 ± 8.28	39.44 ± 4.86	.914
Catheter ablation (n)	5 (33.3%)	37 (35.6%)	1
HATCH score	1 (0–2)	1 (0–1)	.501
Follow-up time, mo	8 (7–11)	11 (7–14)	.113

ACEI = angiotensin converting enzyme inhibitor, ARB = angiotensin receptor blocker, BMI = body mass index, CAD = coronary artery disease, HATCH = hypertension, age, cerebral ischemia event, chronic obstructive pulmonary disease, heart failure, hsCRP = high sensitive C-reactive protein, LA = left atrium, LV dysfunction = left ventricular dysfunction.

## 4 Discussion

Increasing evidence has indicated that atrial fibrillation is closely linked to aging. It is justifiable, therefore, to examine biological indicators of aging to provide a better understanding of the etiology of atrial fibrillation. We chose age-dependent telomere attrition rate in peripheral blood leucocytes as an indicator in the present study. The principal findings were as follows: LTLs were independently associated with history of AF; LTLs were significantly shorter from sinus rhythm to paroxysmal AF to persistent AF in an ordinal way; shorter LTL was an independent risk factor for persistent AF, but not for paroxysmal AF; shorter LTLs could be a promising biomarker in predicting the progression of AF.

Persistent AF is thought to be a more severe type than paroxysmal AF as its longer course of arrhythmia condition and less sensitivity of cardioversion. In 2014, Carlquist et al^[14]^ utilized a cross-sectional analysis strategy and observed a significant association between AF and LTL after adjusting for age and cardiovascular risk factors. After examining the AF subtypes, paroxysmal AF, but not persistent AF, was associated with LTL. Our former research incorporated 277 patients received catheter ablation and found that shorter LTL is an independent risk factor for AF recurrence at 14.20 ± 5.04 months of follow-up (HR = 3.17 [95% CI: 1.23–8.15, *P* = .007]). However, the difference in LTL was not compared between paroxysmal and persistent AF.^[19]^ The current study utilized cross-sectional analysis similar to the Carlquist et al study, and the results agree in part with this study in that we observed a significant association of AF and LTL. As for the AF subtypes, however, we found that shorter telomere length correlated with more severe the type of AF. The disparity between the 2 studies could be Carlquist et al challenged a long-recognized notion that there was a progression from paroxysmal to persistent AF. One possible explanation could be the different selection of the study population. The participants of the former study came from a population that underwent angiography and most of whom had coronary heart disease, thus they had a significant higher rate of coronary artery disease than persistent or permanent AF patients (82.7% versus 78.5% and 65.1%, *P* = .01) which could lead to potential selection bias. Mean LTL could reflect systemic influences on telomere attrition rate, especially the senescent of circulating immune cells,^[20]^ a major pro-inflammatory process. While inflammation has been extensively attributed to the cause of pathogenesis of atrial fibrillation in vivo and in vitro,^[21,22]^ telomere attrition in immune cells (namely leukocyte telomere length shortening in the present study) should be a relevant factor in the etiology of atrial fibrillation. Additionally, atrial fibrillation, especially persistent AF, could induce inflammation and subsequent cardiac remodeling (described as “AF begets AF”),^[23]^ in which telomere attrition causes further attrition.^[20]^ This vicious circle would be likely to cause a shorter LTL in persistent AF patients than in paroxysmal AF patients, as seen in the present study.

Roberts et al,^[15]^ Siland et al,^[16]^ and Staerk et al^[17]^ conducted longitudinal study designs rather than cross-sectional study focusing on the relationship between incidence of AF and LTLs. These 3 prospective studies came to similar conclusions, finding that aging and short LTL were independent risk factor for AF. Although study designs were similar in these 3 studies, their study population varied. In studies by Roberts et al and Siland et al, after inclusion and LTL determination, both groups independently used a considerable sample size of subjects without prevalent AF (n = 1639 and 7775 with an approximately mean age of 72.2 and 48.9, respectively) for >10 years. Although the incidence of AF was found to be much higher by Roberts et al than Siland et al (28.1% vs 4.7%), it likely owed to the older age of the participants, and they found no association between LTL and incidence of AF after a mean follow-up time of 11.6 and 11.4 years when age and other known AF risk factors were accounted for. In the Framingham Heart Study from Staerk et al, the mean follow-up time was 15.1 ± 4.2 years, but only 184 of 1143 (16.1%) participants developed AF and no association between LTL and AF was found. One significant point was the study population in these studies might have been either too young or too old to observe development of AF. The hazard ratio of age in AF underwent a dramatic ascending trend in AF patients aged from 50 to 70.^[24]^ Therefore, it is reasonable to conclude that studies incorporating younger or older subjects will underestimate the influence of telomeres because most individuals would not have developed AF in younger individuals (i.e., age <50) and there is evidence that telomeres exert a milder or inverse influence on cardiovascular diseases in young men^[25]^; the impact of telomeres would be mitigated in much older subjects because of increasingly effects of other AF risk factors.^[26]^ In agreement with these studies, our study did not find significant difference in LTL between PAF and sinus rhythm patients after incorporating multiple factors which are different between the 2 groups. In addition, no newly onset cases of AF were observed in the sinus rhythm group after 18 months of follow-up. However, we did find a significant association between persistency of AF and LTLs. We then found out a cut-off LTL value (*t*/*s* = 0.73) that best distinguished PsAF from PAF based on ROC curve analysis. Using this cut-off value as a grouping factor, we found that the LTLs have a significant role in predicting future persistency from paroxysmal AF within a median follow-up period of 11 months (namely LTL ≤0.73 could help predict progression from paroxysmal AF to persistent AF). These findings indicate that LTL might correlated to AF progression from paroxysmal to persistence. But did not predict new onset of AF in those with normal rhythm. The progression of AF from paroxysmal to persistent increased would significantly enhance the risk for AF-related comorbidities and resistance to AF therapies, such as stroke^[27]^ and AF recurrence after catheter ablation.^[28]^ Therefore, the results of our study potentially provide a new biomarker which could help guide therapeutic decisions and shed light on prognostic evaluation.

## 5 Limitations

The present study suffers from several limitations. First, the results differed in part from the previous study,^[14]^ despite being of a similar sample size, thus further studies and/or meta-analysis are still needed to increase statistical power. Second, the follow-up time was relatively short and the cross-sectional design, we could not conclude any causal relationship or role of possible residual confounding factors. However, we did carry out multiple regression analyses in an attempt to adjust for the known AF risk factors and clinical conditions which differed among the different groups, after which the different LTLs remained significant. In addition, the follow up data lent strength to the role of LTL in predicting progression of AF from paroxysmal to persistent. Third, we determined relative LTL based on a real-time PCR analysis instead of the gold standard Southern blotting, but our method has long been a recognized measure in determining LTL/disease association studies as well. It may be necessary to utilize the absolute length of LTLs as measured by Southern blotting in clinical settings, but this did not undermine the conclusions of the present study. Fourth, despite LA volume is the best measurement for atrial enlargement, its measurement is not a routine procedure, so we could not obtain this data in the present study. Because LA diameter is often utilized for evaluation of atrial enlargement, it could be used as a substitute for LA volume in daily clinical practice. Finally, the follow-up method did suffer from possible omissions, 24-hour Holter monitor was arranged for all 350 patients during followed-up at 3, 6, 12, and 18 months. However, we were unable to discover patients with asymptomatic PAF if they presented with normal ECG and Holter moniter results during follow-up, so the progression and new onset of AF might have be underestimated.

## 6 Conclusion

In conclusion, we observed a significant shorter LTL in patients with AF, and the significance remained in patients with persistent AF, but not paroxysmal AF, after adjustment for other known AF risk factors. In addition, shorter LTLs could be a potential biomarker in predicting persistent AF from paroxysmal AF.

## Author contributions

**Data curation:** Yuanfeng Gao.

**Funding acquisition:** Xinchun Yang.

**Investigation:** Siyu Wang, Lei Zhao, Roumu Hu, Ye Liu.

**Supervision:** Yuanfeng Gao, Lei Zhao, Xinchun Yang.

**Writing – original draft:** Ye Liu.

**Writing – review & editing:** Siyu Wang, Roumu Hu, Xinchun Yang.
